# Lipopolysaccharide-Induced Acute Kidney Injury Is Dependent on an IL-18 Receptor Signaling Pathway

**DOI:** 10.3390/ijms18122777

**Published:** 2017-12-20

**Authors:** Yuji Nozaki, Shoichi Hino, Jinhai Ri, Kenji Sakai, Yasuaki Nagare, Mai Kawanishi, Kaoru Niki, Masanori Funauchi, Itaru Matsumura

**Affiliations:** Department of Hematology and Rheumatology, Kindai University Faculty of Medicine, Osaka-sayama, Osaka 589-8511, Japan; s-hino@med.kindai.ac.jp (S.H.); jinhai@med.kindai.ac.jp (J.R.); kenji-s-101@med.kindai.ac.jp (K.S.); nagare@med.kindai.ac.jp (Y.N.); kazuya-k@med.kindai.ac.jp (M.K.); asuka@med.kindai.ac.jp (K.N.); mn-funa@med.kindai.ac.jp (M.F.); kougen@med.kidai.ac.jp (I.M.)

**Keywords:** acute renal failure, cytokine, CD4^+^ T cells

## Abstract

The proinflammatory cytokine interleukin (IL)-18 is an important mediator of the organ failure induced by endotoxemia. IL-18 (known as an interferon-gamma (IFN-γ) inducing factor), and other inflammatory cytokines have important roles in lipopolysaccharide (LPS)-induced acute kidney injury (AKI). We investigated the effect of inflammatory cytokines and Toll-like receptor 4 (TLR4) expression, an event that is accompanied by an influx of monocytes, including CD4^+^ T cells and antigen-presenting cells (APCs) in IL-18Rα knockout (KO) mice and wild-type (WT) mice after LPS injection. In the acute advanced phase, the IL-18Rα KO mice showed a higher survival rate and a suppressed increase of blood urea nitrogen, increased levels of proinflammatory cytokines such as IFN-γ and IL-18, the infiltration of CD4^+^ T cells and the expression of kidney injury molecule-1 as an AKI marker. In that phase, the renal mRNA expression of the M1 macrophage phenotype and C-C chemokine receptor type 7 as the maturation marker of dendritic cells (DCs) was also significantly decreased in the IL-18Rα KO mice, although there were small numbers of F4/80^+^ cells and DCs in the kidney. Conversely, there were no significant differences in the expressions of mRNA and protein TLR4 after LPS injection between the WT and IL-18Rα KO groups. Our results demonstrated that the IL-18Rα-mediated signaling pathway plays critical roles in CD4^+^ T cells and APCs and responded more quickly to IFN-γ and IL-18 than TLR4 stimulation in the pathogenesis of LPS-induced AKI.

## 1. Introduction

Interleukin (IL)-18 is a proinflammatory cytokine produced by antigen-presenting cells (APCs) and T cells such as macrophages, dendritic cells (DCs) and CD4^+^ T cells. The IL-18 receptor (IL-18R) is a heterodimer comprised of a signaling IL-18Rβ subunit (also called IL-1RAcPL and IL-1R7) and a ligand-binding IL-18Rα subunit. Downstream from IL-18R, the signaling activates interleukin-1 receptor-associated kinase 4 (IRAK4) and the adaptor molecule MyD88, in a scenario similar to that of other IL-1 and Toll-like receptors (TLRs) [1,2,3].

An infection or the exposure to a pathogen such as a bacterium, virus, parasite or fungus can trigger the production of IL-18 by APCs and T cells [4]. IL-18 is used as an early marker of renal tubular damage in acute kidney injury (AKI) in humans [5], and in a mouse model, IL-18 treatment worsened acute tubular necrosis [6,7]. It was demonstrated that IL-18 is also an interferon-gamma (IFN)-γ- and tumor necrosis factor (TNF)-inducing factor based on to its ability to induce the production of TNF and IFN-γ in APCs, T cells and monocytes [8]. We hypothesized that if an injured kidney becomes hyper-responsive to lipopolysaccharide (LPS), which is a component of Gram-negative bacteria, it may increase the levels of circulating inflammatory cytokines such as TNF and IFN-γ and result in a worsening of the AKI, resulting in further damage to renal function.

The reported incidence of AKI ranges from 5% in all hospitalized patients to 30–50% in intensive care units [9]. Infiltrating cells have been indicated to play important roles in the initiation and progress of tubule dysfunction and structural injury in AKI [10] by inhibiting infiltrations by lymphocytes [11] or macrophages [12], leading to decreased tubule damage. In the repair of epithelial cells that occurs in response to an AKI, the differentiation, migration and proliferation of surviving tubular cells is essential for the restoration of tissue integrity [13,14].

We demonstrate herein that when IL-18Rα knockout (KO) mice experienced an LPS-induced AKI, they had markedly ameliorated renal function. The renal status of the IL-18Rα KO mice was also ameliorated by the downregulation of inflammatory cytokines after LPS injection. The expressions of mRNA and protein Toll-like receptor 4 (TLR4) in IL-18Rα KO mice did not differ significantly between before and after they were injected with LPS. Our results indicate that in the LPS-induced AKI model mice, IL-18Rα has a crucial and direct signaling pathway in CD4^+^ T cells in APCs and not via a TLR4 signaling pathway.

## 2. Results

### 2.1. Interleukin (IL)-18Rα Deficiency Improves Survival after Lipopolysaccharide (LPS)-Induced Acute Kidney Injury (AKI)

**Figure 1:**
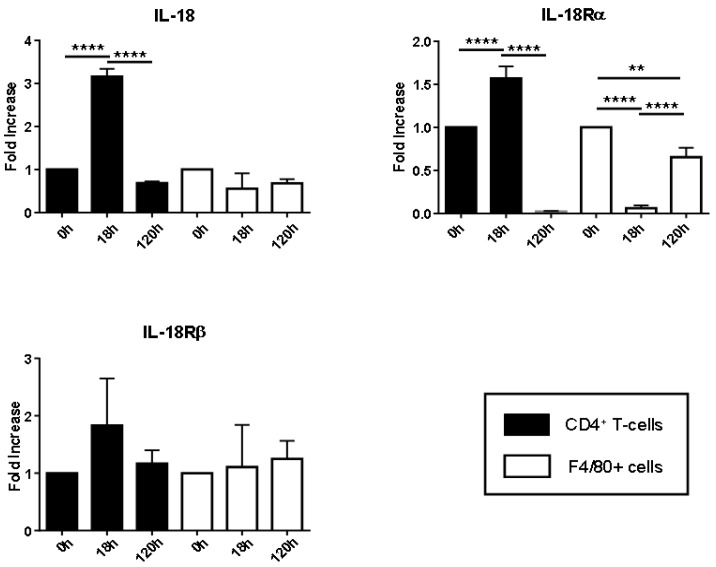
Lipopolysaccharide (LPS) affects the expression levels of interleukin (IL)-18, IL-18Rα and IL-18Rβ in the mouse kidney. C57BL/6 mice were injected intraperitoneally with 30 mg/kg LPS and sacrificed at 18 and 120 h after LPS injection. At 18 and 120 h, CD4^+^ T cells and F4/80^+^ cells were isolated using a BD FACSAria^TM^ special-order research product with purities of 90–95% from splenocytes in C57BL/6 WT mice. Gene expressions of IL-18, IL-18Rα and IL-18Rβ were measured by real-time PCR. In each experiment, the expression levels were normalized to the expression of 18SrRNA and were expressed relative to the values of saline-treated control mice. The data are the mean fold-increase ± SEM: ** *p* < 0.01, **** *p* < 0.0001, the mice without and with LPS injection at 0 and 120 h (*n* = 4 and *n* = 6) vs. 18 h (*n* = 4) after LPS injection.

To determine whether an LPS-induced AKI stimulates the IL-18/IL-18R signaling pathway via CD4^+^ T cells and macrophages, we measured the mRNA expressions of IL-18, IL-18Rα and IL-18Rβ in isolated splenic CD4^+^ T cells and F4/80^+^ cells using fluorescence-activated cell sorting (FACS) in wild-type (WT) C57BL/6 mice before and after LPS injection (Figure 1).

In the CD4^+^ T cells, the IL-18 and IL-18Rα mRNA expressions peaked at 18 h. The IL-18Rα mRNA expression also peaked at 18 h, but it did not reach a significant increase, unlike those at 0 and 120 h. In the F4/80^+^ cells, the IL-18 mRNA expression did not differ significantly between before and after the LPS injection. The IL-18Rα mRNA expression was significantly reduced at 18 h compared to the levels at 0 and 120 h. After the downregulation, the IL-18Rα mRNA expression at 120 h was increased significantly compared to that at 18 h. Interestingly, the IL-18Rβ mRNA expression did not differ significantly between before and after the LPS injection.

**Figure 2:**
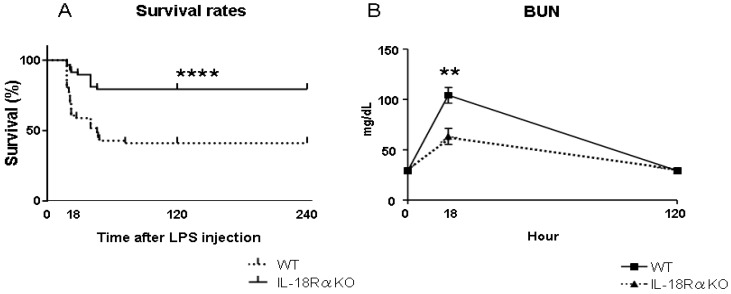
Effect of IL-18Rα on the survival and blood urea nitrogen (BUN) level in the development of AKI after LPS injection. (**A**) Survival of WT and IL-18Rα KO mice subjected to sepsis by LPS injection. Mice were evaluated 2×/day until 120 h post-LPS injection (WT *n* = 13; IL-18Rα KO mice *n* = 18; **** *p* < 0.001). (**B**) Renal function of WT and IL-18Rα KO mice at 0, 18 and 120 h before and after LPS injection, assessed by BUN levels. The data are the mean values ± SEM (** *p* < 0.01, WT vs. IL-18Rα KO mice).

We next observed that the IL-18Rα KO mice had a significantly higher survival rate at 120 h (WT 46.2% vs. IL-18Rα KO 88.9%) (Figure 2A). The LPS-induced rise in blood urea nitrogen (BUN) levels at 18 h was also significantly decreased in the IL-18Rα KO mice (WT 104.1 ± 7.7 vs. IL-18Rα KO 63.4 ± 8.0 mg/dL) (Figure 2B). At 120 h, the BUN levels in all mice were decreased to the same level as that before LPS injection (WT 29.3 ± 0.3 vs. IL-18Rα KO 29.5 ± 0.9 mg/dL).

### 2.2. Serum Biomarkers in Acute Kidney Injury

**Figure 3:**
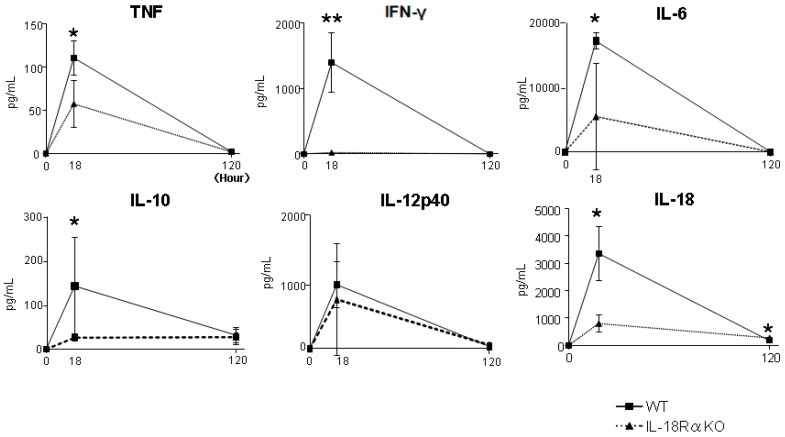
Effect of IL-18Rα on the production of inflammatory cytokines after LPS injection. Serum TNF, IFN-γ, IL-6, IL-10, IL-12p40 and IL-18 productions were measured as biomarkers of AKI at 0 (KO and WT, *n* = 3), 18 (*n* = 11 and *n* = 10) and 120 h (*n* = 16 and *n* = 6) before and after LPS injection. The data are the mean values ± SEM (* *p* < 0.05, ** *p* < 0.01, WT vs. IL-18Rα KO mice).

The serum levels of TNF, IFN-γ, IL-6, IL-10, IL-12p40 and IL-18 were measured as biomarkers in AKI (Figure 3). These cytokine levels peaked at 18 h and were greatly decreased at 120 h after LPS injection. The serum TNF, IFN-γ, IL-6, IL-10 and IL-18 levels at 18 h were significantly decreased in the IL-18Rα KO mice compared to the corresponding levels in the WT mice, as follows (IL-18Rα KO vs. WT for all): TNF, 57.8 ± 27.1 vs. 111.2 ± 19.6 pg/mL; IFN-γ, 22.8 ± 15.1 vs. 1397.2 ± 459.1 pg/mL; IL-6, 5.5 ± 3.3 vs. 15.1 ± 2.1 ng/mL; IL-10, 15.6 ± 6.8 vs. 143.1 ± 49.7 pg/mL; and IL-18, 799.0 ± 326.2 vs. 3362.0 ± 979.2 pg/mL.

### 2.3. The Infiltration of CD4^+^ T Cells and Antigen-Presenting Cells (APCs) in the Kidney

**Figure 4:**
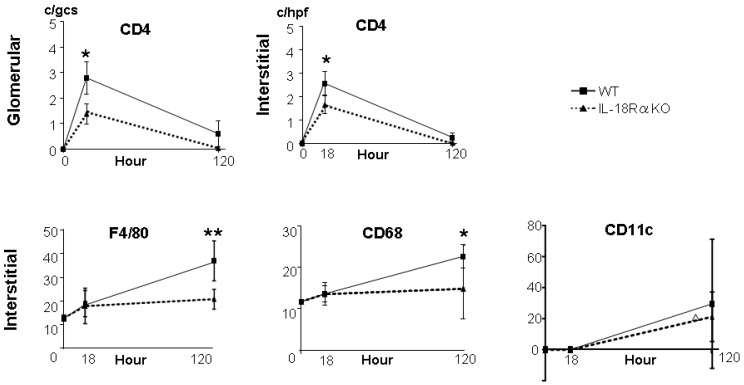
Effect of IL-18Rα on the accumulation of inflammation cells in the glomerulus and interstitium after LPS injection The accumulation of CD4^+^ T cells, F4/80^+^, CD68^+^ and CD11c^+^ cells in the glomerulus and interstitium at 0 (KO and WT, *n* = 3), 18 (*n* = 11 and *n* = 10) and 120 h (*n* = 16 and *n* = 6) before and after LPS injection. The data are the mean ± SEM (* *p* < 0.05, ** *p* < 0.01, the cell numbers in IL-18Rα KO vs. WT mice).

We evaluated the infiltration of CD4^+^ T cells and APCs (i.e., inflammation cells) in the renal glomerulus and interstitium before and after LPS injection (Figure 4). The infiltration of CD4^+^ T cells peaked at 18 h, whereas the numbers of APCs increased gradually to 120 h after LPS injection. The glomerular CD4^+^ T cell numbers in the IL-18Rα KO mice were significantly decreased compared to those in the WT mice at 18 and 120 h (WT 2.8 ± 0.6 vs. IL-18Rα KO 1.1 ± 0.3 c/gcs, * *p* < 0.05). The numbers of interstitial macrophages (i.e., F4/80^+^ and CD68^+^ cells) in the IL-18Rα KO mice were significantly decreased at 120 h (F4/80^+^; WT, 37.0 ± 4.2 vs. IL-18Rα KO, 20.8 ± 1.9 and CD68^+^; 22.2 ± 1.7 vs. 17.6 ± 1.1 c/hpf, * *p* < 0.05). The numbers of interstitial DCs (CD11^+^ cells) showed no significant differences between the WT and IL-18Rα KO groups at 120 h (WT 29.6 ± 13.9 vs. IL-18Rα KO 21.3 ± 4.6 c/hpf). There were no infiltrations of F4/80^+^, CD68^+^ or CD11c^+^ cells in the glomerulus (Supplementary Figure S1).

### 2.4. Renal Kim-1 Expression

**Figure 5:**
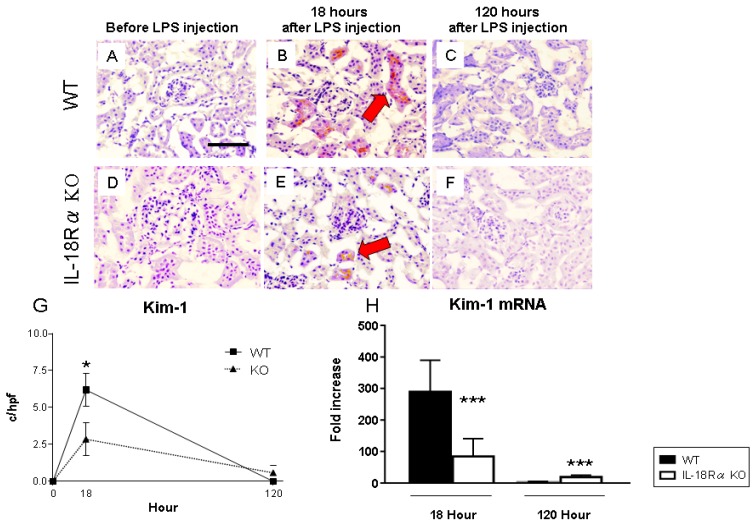
The effect of IL-18Rα on the Kim-1 expression after LPS injection. No tubular Kim-1 was observed in the WT or IL-18Rα KO mice without LPS injection (**A**,**D**; *n* = 3). At 18 h post-LPS injection, there were more tubular Kim-1^+^ cells (indicated by **red arrows** at high power, ×400) in the WT mice (**B**, *n* = 11) than in the IL-18Rα KO mice (**E**, *n* = 10). At 120 h after LPS injection, few tubular Kim-1 cells are present in the WT and IL-18Rα KO mice (**C**, *n* = 16; **F**, *n* = 6). In the IL-18Rα KO mice, there were significantly fewer Kim-1^+^ proximal tubules (**G**). At 18 h, the intrarenal Kim-1 mRNA expression was decreased in the IL-18Rα KO mice compared to the WT mice (WT, *n* = 11; KO, *n* = 10). However, the Kim-1 mRNA expression was increased in the IL-18Rα KO mice compared to the WT mice at 120 h after LPS injection (**H**, *n* = 16 and *n* = 6). Photomicrographs were taken at ×400. Values are the mean ± SEM (* *p* < 0.05 and *** *p* < 0.005). Scale bar, 50 μm.

Figure 5 shows the tubular Kim-1 expression before and after LPS injection. In the mice without LPS injection, Kim-1 was not detected in the kidney (Panels A, D). There were more of these cells in the tubules at 18 h after LPS injection (Panels B, E). At 18 h, the Kim-1^+^ cell numbers in the IL-18Rα KO mice were significantly decreased compared to those in the WT mice (WT 6.2 ± 1.1 vs. IL-18Rα KO 2.7 ± 1.1 c/hpf) (Panel G). In the mice at 120 h after LPS injection, the Kim-1^+^ cell numbers were dramatically decreased in both groups. The Kim-1^+^ cell numbers in the IL-18Rα KO mice tended to be increased at 120 h (IL-18Rα KO 0.6 ± 0.5 vs. WT 0.0 ± 0.0 c/hpf). At 18 h, the Kim-1 mRNA expression in the IL-18Rα KO mice (84.8 ± 22.7-fold increase) was significantly decreased compared to that in the WT mice (289.6 ± 37.8-fold increase; panel H). Conversely, at 120 h, the Kim-1 mRNA expression in the IL-18Rα KO mice (19.5 ± 2.4-fold increase) was significantly increased compared to that in the WT mice (3.2 ± 1.0-fold increase).

### 2.5. Renal mRNA Expression in LPS-Induced AKI

Inflammatory cytokines and chemokines are well documented in LPS-induced AKI [8], and we therefore measured the mRNA expressions in the kidney by conducting RT-PCR (Table 1). There were widespread reductions in the cytokines and chemokine mRNA expression (IL-6, -10, -18, IFN-γ and TNF) in the IL-18Rα KO mice at 18 h. At 120 h, these cytokines were downregulated and showed no significant differences between the WT and IL-18Rα KO groups. Notably, the IL-12 mRNA expression did not reach significance in the two groups. Some studies reported that IL-12 is a Th-1-promoting and proinflammatory cytokine that promotes the production of IFN-γ from T and NK cells, particularly in the presence of IL-18 [15,16]. Here, we assessed the expression of mRNA for two Th-cell subset transcription factors, T-bet (Th1) and GATA3 (Th2), in renal mRNA from each mouse group. The T-bet expressions at 18 h after the LPS injection were reduced in the IL-18Rα KO mice compared to those in the WT mice, but the GATA3 expression was not changed in either group. Therefore, IL-18Rα deficiency shifted systemic responses away from the Th1 phenotype.

### 2.6. Toll-Like Receptor 4 (TLR4) Expression in LPS-Induced AKI

**Figure 6:**
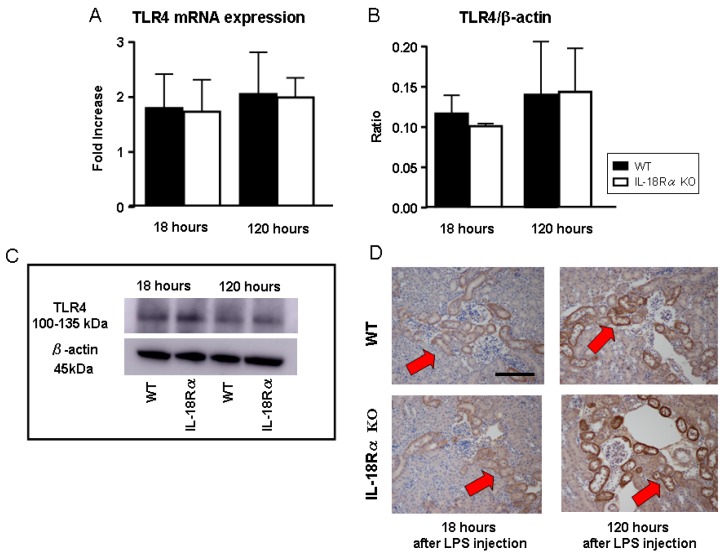
Effect of IL-18Rα on TLR4 in LPS-induced AKI. The gene expression (**A**), protein levels (**B**) and tubular staining of TLR4 (**D**) in WT and IL-18Rα KO mice at 18 (*n* = 11, *n* = 10) and 120 h (*n* = 16, *n* = 6) after LPS injection were assayed by real-time PCR, Western blotting and immunohistochemistry. (**C**) A representative band of TLR4 and β-actin. In the immunohistochemical staining (**D**), TLR4^+^ cells were present in the dilated tubules, indicated by **red arrows** (×400) in the WT and IL-18Rα KO mice at 18 and 120 h after LPS injection. The data are the mean ± SEM. Photomicrographs: ×400. Scale bar, 50 μm.

Figure 6 shows the mRNA expression, protein levels and tubular staining of renal TLR4 after LPS injection by real-time PCR, Western blotting and immunohistochemistry. There were no significant between-group differences in the mRNA expression or protein levels of TLR4 after LPS injection. At 18 h, tubular TLR4^+^ cells were already present in the dilated tubules in both the WT and IL-18Rα KO groups. The number of tubular TLR4^+^ cells was not increased in either group at 120 h after LPS injection. There were no significant differences in the two groups after LPS injection.

### 2.7. Intracellular Interferon-Gamma (IFN-γ) Staining in CD4^+^ T Cells and APCs after LPS-Induced AKI

IFN-γ has an important role in the response to LPS [17]. We also observed the strong reduction of the serum levels and renal mRNA expression of IFN-γ in the IL-18Rα KO mice in the present study. Table 2 provides the results of IFN-γ^+^ staining of intracellular CD4^+^ T cells and the FACS analysis of F4/80^+^, CD11b^+^ and CD11c^+^ as APCs. At 18 h, the IFN-γ^+^ staining in the CD4^+^ T cells, F4/80^+^ and CD11b^+^ cells in the IL-18Rα KO mice was significantly decreased compared to that in the WT mice. In addition, there were no significant differences in IFN-γ^+^ staining in these cells at 120 h after LPS injection. IFN-γ^+^ in CD11c^+^ cells in the two groups was not changed at 18 or 120 h.

### 2.8. Classically- and Alternatively-Activated Macrophages in LPS-Induced AKI

We next quantified the renal expression of M1 or M2 macrophage phenotypes and C-C chemokine receptor type 7 (CCR7) by real-time PCR (Table 3). The upregulation of CCR7 is associated with the maturation of DCs [18]. In response to LPS-induced AKI, there were increases in a renal M1 marker nitric oxide synthase (iNOS), the M2 marker (IL-4Rα) and the M1/M2 ratio that were higher at 18 than 120 h after LPS injection. At 18 h, there were significant decreases in the mRNA of the M1 marker iNOS, the M2 marker IL-4Rα and the M1/M2 ratio in the IL-18Rα KO mice. There was also a significant decrease in the CCR7 mRNA level in the IL-18Rα KO mice at 18 h.

### 2.9. Splenocyte Adoptive Transfer Restored the Kidneys’ Susceptibility to LPS-Induced AKI

**Figure 7:**
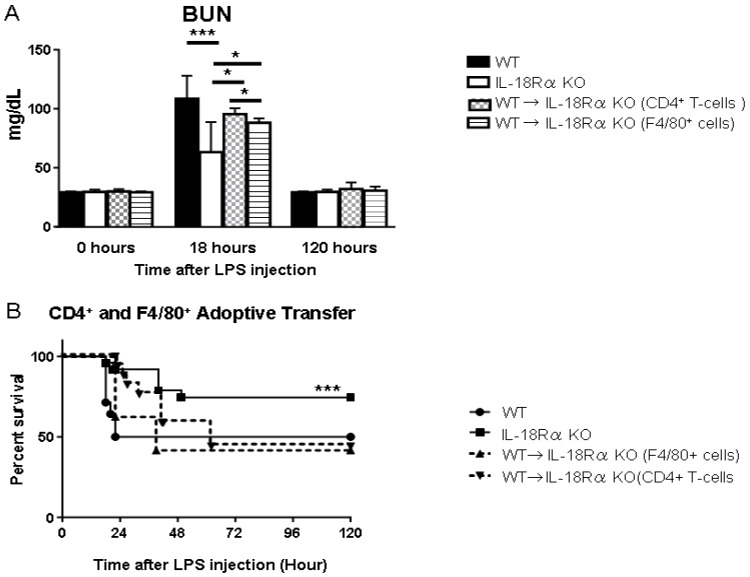
Effect of splenocyte transfer on renal function and survival rates. (**A**) CD4^+^ T cells and F4/80^+^ cells (2 × 10^6^/mouse) from splenocytes of C57BL/6 WT mice were injected intravenously into each IL-18Rα KO mouse two days before LPS injection. Mice were sacrificed at 0 (each group; *n* = 3), 18 (*n* = 5 and *n* = 9) and 120 h (*n* = 5 and *n* = 7) after LPS injection. The data are the mean ± SEM (* *p* < 0.05, *** *p* < 0.005). (**B**) The survival of the WT mice, the IL-18Rα KO mice and the IL-18Rα KO mice that received a transfer of CD4^+^ T cells or F4/80^+^ cells subjected to sepsis by LPS injection. Mice were evaluated 2×/day until 120 h (*** *p* < 0.005). As controls, IL-18Rα KO and WT mice were also culled at 0 (each group; *n* = 3), 18 (*n* = 10 and *n* = 11) and 120 h (*n* = 5, *n* = 4), and we determined the survival rate at 120 h as an endpoint.

Monocytes have been implicated as a key factor in the development of systemic inflammation during sepsis [19]. To determine whether IL-18Rα in CD4^+^ T cells and F4/80^+^ cells as APCs affects the renal function and survival in LPS-induced AKI, we transferred splenocytes from normal WT mice into IL-18Rα KO mice (Figure 7). At 18 h, the renal function of the IL-18Rα KO mice that received a transfer of CD4^+^ T cells and F4/80^+^ cells from WT mice showed more severe AKI, manifested by a remarkable rise in BUN, compared to IL-18Rα KO mice: WT 108.6 ± 5.9; IL-18Rα KO (that received CD4^+^ T cells) 95.4 ± 2.3; and IL-18Rα KO (with F4/80^+^ cells) 88.1 ± 1.4 vs. IL-18Rα KO 63.4 ± 8.0 mg/dL. These results were supported by the survival rate in the WT mice, IL-18Rα KO mice and the IL-18Rα KO mice that received a transfer of CD4^+^ T cells and F4/80^+^ cells mice at 22 h after the LPS injection: IL-18Rα KO 92% vs. WT 50%, IL-18Rα KO (with CD4^+^ T cells) 62.5% and IL-18Rα KO (with F4/80^+^ cells) 100%. At 120 h, we observed that the IL-18Rα KO mice had the highest survival rate compared to the other groups: IL-18Rα KO 74.5% vs. WT 50%, IL-18Rα KO (with CD4^+^ T cells) 41.7% and IL-18Rα KO (with F4/80^+^ cells) 44.1% (Figure 7B).

## 3. Discussion

We have published several studies about IL-18Rα. For example, we showed that MRL- Fas^lpr^mice (well known as lupus model) cross-bred with mice deficient in IL-18Rα had a better survival rate and lessened nephritis due to reduced levels of autoantibodies [20]. We then observed that IL-18Rα may mediate anti-inflammatory responses through suppressors of cytokine signaling-1 and/or -3 in cisplatin-induced AKI [21]. Our later investigation showed that IL-18Rα also mediates apoptosis in ischemia/reperfusion injury model mice [7]. It remains unknown how APCs such as macrophages and DCs affect the immune response in the acute advanced and recovery phases. Our present findings demonstrate that the numbers of inflammatory cells in the kidney were decreased and the renal function was ameliorated in IL-18Rα-deficient mice after LPS injection.

Studies from 1997 and 200 demonstrated that the AKI induced by LPS or by other toxins results in the release of inflammatory mediators, even without lowering blood pressure [22,23]. In the present study, the blood pressure values of the mice as measured by a tail cuff were not significantly lower post-LPS injection (Supplementary Table S1). We have sought to determine the mechanisms underlying the activation of IL-18Rα and IL-18Rα signaling in the immune system’s response to LPS-induced AKI. To gain a better understanding of these issues, we thus investigated the effects of the inflammatory cytokines and TLR4 expression, which is accompanied by the influx into the kidney of monocytes such as APCs and CD4^+^ T cells in the acute advanced and recovery phases.

Honda et al. showed that CD4^+^ T cells express surface TCRs (T-cell receptors) that identify foreign antigenic peptides bound to MHC (major histocompatibility complex) molecules on the surface of APCs in the lymph nodes [24]. These naive T cells are induced to proliferate rapidly by signaling via the TCR and then integrate additional signals that allow them to differentiate into Th1, Th2 or Th17 phenotypes [23,25,26]. Of particular interest is the report that Th1 cells can gain the capacity to secrete IFN-γ (which is a key cytokine in the activation of macrophages and host protection against intracellular pathogens) and IL-18 [27,28]. After effector CD4^+^ T cells recognize peptide/MHC complexes displayed on tissue APCs [29,30], they can secrete cytokines, and the inflammatory cytokine production is thus targeted locally to the precise anatomical site of infection. Notably, IFN-γ and IL-18 elicited from Th1 cells can also activate neighboring macrophages [22,31], providing some nonspecific defense in the area around the Th1 stimulation.

Calvani et al. noted that IL-18 apparently mediates inflammation by initiating and expanding Th1 responses and by its direct effects on lymphocytes and macrophages [27]. IL-18 is nephritogenic; it recruits IL-18R-positive DCs to the kidneys, enhancing the immune-mediated renal damage [32,33]. When IL-18 binds to its specific receptor IL-18R, recruiting the adaptor molecule MyD88 to the Toll/IL-1R domain of IL-18R, IL-18 can thereby induce the production of other inflammatory cytokines (e.g., IFN-γ, TNF-α, IL-6 and IL-1β) [34]. There are several cytokine-deficient mouse strains that are resistant to the lethal actions of LPS [35]. For example, Okamura et al. reported that IFN-γR KO mice displayed enhanced resistance to LPS, and the severity of the clinical changes caused by LPS (e.g., liver injury, weight loss) was greatly decreased in the mice [36].

However, IFN-γ production can itself be modulated by regulatory cytokines such as IL-12 and -18. IL-12 can both induce IFN-γ production and act synergistically with IL-18 [37]. IFN-γ production by B cells and macrophages occurred only after simultaneous treatment with IL-12 and -18 [38,39], whereas it was reported that T cells and natural killer (NK) cells do not require either IL-12 or IL-18 to produce IFN-γ [29,40]. Unusually high levels of IL-18 (which can induce IFN-γ production) and TNF-α have been described in humans [41]. Such IL-18 levels are associated with an imbalance between the natural inhibitor of IL-18 (i.e., IL-18 binding protein [IL-18BP]) and IL-18 that is followed by an excess of free IL-18 [42]. The IL-18BP promoter has an element that is responsive to IFN-γ, and IFN-γ can effectively induce the production of IL-18BP [43].

Using a murine model of hemophagocytic lymphohistiocytosis treated with IL-18BP, Chiossone et al. reported that this treatment decreased hemophagocytosis and reversed both liver and spleen damage in the mice [44]. In addition, treating the mice with IL-18BP reduced the production of both IFN-γ and TNF-α by CD8^+^ T cells and NK cells, and it reduced the expression of Fas ligand on the surfaces of NK cells. In the present study, we investigated the effects of inflammatory cytokines and TLR4 expression (which is accompanied by an influx of monocytes including CD4^+^ T cells and APCs) in IL-18Rα-deficient mice after LPS injection, and we observed that the survival rate and renal function were improved by suppressing serum inflammatory cytokines and the renal cytokine mRNA expressions (especially those of IL-18, IFN-γ, TNF and IL-6) and an accumulation of CD4^+^ T cells in IL-18Rα KO mice. It is possible that the administration of IL-18BP may eventually be revealed to have potential in the therapy of AKI in endotoxemia.

To understand the mechanisms underlying the IL-18R signaling pathway when mice are exposed to LPS-induced AKI, we used IL-18Rα-deficient mice in this study. Specific cell-surface receptors mediate the binding of IL-18 to its target cells; this is similar to the IL-1R mechanism. An α-chain (IL-18Rα) [45,46] and a β-chain (IL-18Rβ or AcPL) comprise the receptor of IL-18 [47]. Born et al. proposed that IL-18Rβ does not itself interact directly with IL-18; they also suggested that IL-18Rα is responsible for the IL-18 binding. We investigated whether an IL-18Rβ signaling pathway contributes to decreased renal function. We treated WT mice with an anti-IL-18Rβ antibody in a cisplatin-induced renal injury model [21], and we found that this treatment reduced the renal function. The involvement of an IL-18Rβ signaling pathway in the immune systems in AKI model mice remains unknown. Further studies of IL-18Rβ-deficient mice with LPS-induced AKI may clarify this mechanism.

At this time, our data suggest that an IL-18Rα-mediated signaling pathway, not via a TLR4 signaling pathway, in CD4^+^ T cells and APCs plays a major part in the immune response to LPS-induced AKI in the acute advanced phase. In the recovery phase, the IL-18Rα-mediated signaling pathway in APCs, especially macrophages, has a critical role in the recovery of the damaged kidney tissue by the clearance of debris in response to LPS-induced AKI. Our present findings also demonstrated that the expressions of IL-18 and IL-18Rα mRNA in CD4^+^ T cells and the IL-18R1^+^ in CD4^+^ T cells and CD11c^+^ T cells peaked at 18 h as the acute advanced phase. Moreover, the survival rate and renal function were ameliorated by suppressing the serum inflammatory cytokines and the renal cytokine mRNA expressions (especially those of IL-18, IFN-γ, TNF and IL-6) and by the suppression of an accumulation of CD4^+^ T cells in IL-18Rα KO mice at 18 h.

Conversely, F4/80^+^, CD69^+^ and CD11c^+^ cells as APCs still showed increased infiltrations regardless of the renal function, and the levels of inflammatory cytokines were normal at 120 h in the recovery phase. These data suggest that F4/80^+^, CD69^+^ and CD11c^+^ cells might have important roles in the internalization and the clearance of apoptotic cells. The necrotic debris in the recovery phase was highlighted by splenocytes’ adoptive transfer from wild-type mice to IL-18Rα KO mice.

In summary, our results demonstrated that an IL-18Rα-mediated signaling pathway plays a critical role in CD4^+^ T cells and APCs and was faster at responding to IFN-γ and IL-18 than TLR4 stimulation in the pathogenesis of LPS-induced AKI. We conclude that as the pathological mechanism, mainly CD4^+^ T cells and APCs in the advanced phase have the critical role in the immune response to LPS-induced AKI.

In summary, our results demonstrated that IL-18Rα-mediated signaling plays critical roles in CD4^+^ T-cells and APCs and was markedly faster at responding to IFN-γ and IL-18 than TLR4 stimulation in the pathogenesis of LPS-induced acute kidney injury. We conclude that mainly CD4^+^ T-cells and APCs in the advanced phase play a direct and critical role in the immune response to the pathological mechanism in LPS-induced acute kidney injury.

## 4. Materials and Methods

### 4.1. Ethics Statement

The animal protocols were approved by the Kindai University Animal Care Committee and were performed in accordance with the Kindai University Animal Care Guidelines (KAME-22-014, 1/4/2010).

### 4.2. Animals

IL-18Rα-deficient (IL-1Rrp^−/−^) C57BL/6 mice were kindly provided by Dr. Shizuo Akira (Osaka University, Osaka, Japan). The C57BL/6 mice used as a wild-type control were purchased from Shizuoka Laboratory Animal Centre (Shizuoka, Japan). All mice were maintained in our specific pathogen-free animal facility. Blood pressure was measured by tail cuff (BP-98A, Softron Japan, Tokyo, Japan) before and 18 and 120 h after the LPS injection. The blood pressure of each mouse was determined as the median of several measurements [48].

### 4.3. Murine Model of Endotoxin-Induced Acute Kidney Injury

In all experiments, 8–10-week-old male mice were injected intraperitoneally with 30 mg/kg LPS (*Escherichia coli* O111:B4, Sigma-Aldrich, St. Louis, MO, USA). Blood was collected in heparinized tubules for the measurement of blood urea nitrogen, TNF, IFN-γ, IL-6, IL-12, IL-10 and IL-18. Mice were sacrificed at 0 (KO and WT; *n* = 3), 18 (*n* = 11 and 10) and 120 h (*n* = 16 and 6) after induction of the AKI with the collection of blood as described above and the harvesting of kidney and spleen tissue. The resulting lethality was monitored for 120 h after LPS injection. BUN was measured by an autoanalyzer (Hitachi, Tokyo, Japan).

### 4.4. Assessment of Renal Injury

Immunohistochemical staining for CD4^+^ T cell, CD68^+^ macrophages as APCs and tubular Kim-1 and TLR4 was performed on 6-μm periodate lysine paraformaldehyde-fixed sections [21]. CD11c^+^ and F4/80^+^ cells as APCs (DCs, CD11c^+^ and pan-macrophages, F4/80^+^ cells) were identified in 4-ìm-thick formalin-fixed sections. The numbers of these cells were assessed in 10 fields per slide at ×400 magnification, and the results are expressed as cells per high-power field (c/hpf). The primary monoclonal antibodies used were rat monoclonal antibody GK1.5 for CD4^+^ T cells (Pharmingen, San Diego, CA, USA), for APCs with CD68^+^ (Serotec, Oxford, U.K.), F4/80 hybridoma culture supernatant (HB 198; American Type Culture Collection, Manassas, MD, USA) and mouse monoclonal antibody for CD11c^+^ (Abcam, Cambridge, U.K.), rabbit polyclonal antibody TLR4 (Novus Biologicals, Littleton, CO, USA) and rat monoclonal antibody Tim-1 (R & D Systems, Minneapolis, MN, USA).

### 4.5. Measurements of mRNA Expression in the Kidney by Real-Time PCR

For the measurement of the mRNA expressions of TNF, the chemokine (C-C motif) ligand 2/monocyte chemoattractant protein-1 (CCL2/MCP-1), GATA3, T-bet and 18SrRNA by FastStart DNA Master Sybr Green I (Applied Biosystems, Foster City, CA, USA) and IFN-γ, IL-4, IL-6, IL-10, IL-12p40, IL-18, Kim-1, TLR4, CCR7, IL-18R1 (encoding IL-18Rα), IL-18R2 (encoding IL-18Rβ), inducible nitric oxidase synthase (iNOS), IL-4Rα and 18SrRNA by TaqMan gene (Applied Biosystems) on whole kidney tissue and splenocytes, we performed a real-time PCR as described [21]. The sequences of the primer and gene database numbers are listed in Table 4 and Table 5. The relative amount of mRNA was calculated using the comparative *C*_t_ (*∆∆C*_t_) method. All specific amplicons were normalized against 18SrRNA, which was amplified in the same reaction as an internal control using commercial reagents (Applied Biosystems) and is expressed as the fold-increase relative to saline-treated control mice.

### 4.6. Serum Cytokines Quantitation by ELISA

Serum TNF, IFN-γ, IL-6, IL-10, IL-12 and IL-18 levels were determined by an enzyme-linked immunosorbent assay (ELISA) kit for each cytokine (BD Biosciences, San Diego, CA, USA) as described [21,49].

### 4.7. Western blotting

Proteins were extracted by homogenization of the kidney at 18 and 120 h after LPS injection in tissue protein extraction reagent (Pierce, Rockford, IL, USA) to determine TLR4, as described [21]. Monoclonal anti-β-actin antibody was obtained from Santa Cruz (St Louis, MO, USA). Anti-mouse TLR4 antibody was obtained from Cell Signaling Technology (Danvers, MA, USA). Peroxidase-conjugated goat immunoglobulin G was purchased from Santa Cruz.

### 4.8. FACS Analysis

For the assessment of intracellular cytokine by a FACSCanto II (Becton Dickinson, Lincoln Park, NJ, USA), we obtained splenocytes at 18 and 120 h after LPS injection. Splenocytes were stained with mAbs for intracellular staining (anti-IFN-γ) conjugated to phycoerythrin (PE). We assessed the intracellular cytokines by determining their percentages in CD4^+^ T cells and F4/80^+^, CD11b^+^ and CD11c^+^ cells.

### 4.9. Cell Sorting and Adoptive Transfer Experiments

CD4^+^ T cells and F4/80^+^ cells were isolated using a BD FACSAria special-order research product (Becton Dickinson) for purities of 90–95% from splenocytes in C57BL/6 WT mice for the real-time PCR and adoptive transfer. Approximately 2 × 10^6^ CD4^+^ T cells and F4/80^+^ cells were injected intravenously into each IL-18Rα KO mouse 2 days before the 30 mg/kg LPS injection. Mice that received a transfer of CD4^+^ T cells and F4/80^+^ cells were sacrificed at 0 (each group; *n* = 3), 18 (*n* = 5 and 9) and 120 h (*n* = 5 and 7) after LPS injection. As controls, IL-18Rα KO and WT mice were also culled at 0 (each group; *n* = 3), 18 (*n* = 10 and 11) and 120 h (*n* = 5 and 4). We determined the survival rate at 120 h of each group of mice as the endpoint.

To confirm T cell reconstitution in IL-18Rα KO mice, we analyzed the splenic mRNA expression of IL-18R1 (encoding IL-18Rα) in recipient mice at 0, 18 and 120 h (Supplementary Figure S2). The flow cytometry antibodies were FITC-anti-CD4, PE-Cy7-anti-F4/80, BV421-anti-CD11b, APC-Cy7-CD11c and PE-anti-IFN-γ (BD Bioscience). For all markers, an isotype-matched irrelevant mAb was used. Cells fluorescing at levels above the negative control were considered positive.

### 4.10. Statistical Analysis

Results are expressed as the mean ± SEM. Groups were compared by the unpaired *t*-test or by an analysis of variance (ANOVA) when more than two groups were compared. Probability values <0.05 were accepted as significant. The survival time was estimated using the Kaplan–Meier method. The log-rank test was used to compare survival times between groups.

## Figures and Tables

**Table 1:** Effect of IL-18Rα on gene expression in LPS-induced AKI.

	18 h	120 h
Cytokines	WT vs. IL-18Rα KO	WT vs. IL-18Rα KO
IL-6	643.2 ± 201.5 vs. 72.1 ± 49.3 **	7.5 ± 4.6 vs. 2.4 ± 0.4
IL-10	76.3 ± 10.3 vs. 33.3 ± 10.6 *	8.4 ± 4.0 vs. 7.3 ± 1.8
IL-12	3.2 ± 0.7 vs. 1.4 ± 0.8	0.3 ± 0.2 vs. 0.3 ± 0.1
IL-18	2.7 ± 0.4 vs. 1.3 ± 0.2 **	1.3 ± 0.4 vs. 1.7 ± 0.1
IFN-γ	4.3 ± 2.5 vs. 1.8 ± 0.4 *	3.4 ± 1.6 vs. 4.6 ± 0.6
TNF	27.5 ± 4.6 vs. 11.9 ± 4.2 *	2.3 ± 0.5 vs. 2.9 ± 0.1
**Chemokines**		
CCL2/MCP-1	59.8 ± 15.7 vs. 28.7 ± 6.7	4.2 ± 1.3 vs. 4.7 ± 0.6
**Th Cell Subset Transcription Factors**		
GATA3	0.8 ± 0.4 vs. 0.9 ± 0.2	1.6 ± 0.7 vs. 1.8 ± 0.5
T-bet	2.5 ± 0.5 vs. 1.0 ± 0.2 *	4.9 ± 1.8 vs. 5.8 ± 0.7

The gene expressions of cytokines, chemokines and Th cell subset transcription factors were measured in IL-18Rα KO and WT mice at 18 and 120 h after LPS injection by real-time PCR. In each experiment, the expression levels were normalized to the expression of 18SrRNA and are expressed relative to the values of saline-treated control mice. The data are the mean fold-increase ± SEM (* *p* < 0.05, ** *p* < 0.01). CCL2/MCP-1, chemokine (C-C motif) ligand 2/monocyte chemoattractant protein-1.

**Table 2:** Intracellular IFN-γ staining in CD4^+^ T cells and APCs after LPS-induced AKI.

IFN-γ^+^	18 h	120 h
	WT vs. IL-18Rα KO	WT vs. IL-18Rα KO
CD4^+^	0.7 ± 0.1 vs. 0.3 ± 0.1 *	0.2 ± 0.1 vs. 0.3 ± 0.1
F4/80^+^	0.2 ± 0.0 vs. 0.1 ± 0.0 *	1.4 ± 0.8 vs. 1.1 ± 0.5
CD11b^+^	0.6 ± 0.1 vs. 0.2 ± 0.0 **	0.1 ± 0.0 vs. 0.2 ± 0.1
CD11c^+^	0.3 ± 0.1 vs. 0.3 ± 0.1	0.6 ± 0.6 vs. 0.6 ± 0.2

The percentage of IFN-γ^+^ in CD4^+^ T cells, F4/80^+^, CD11b^+^ and CD11c^+^ cells was measured by FACS analysis. The data are the mean ± SEM (* *p* <0.05, ** *p* < 0.01).

**Table 3:** M1/M2 macrophages phenotype by real-time PCR.

	18 h	120 h
**M1 Macrophage**	**WT vs. IL-18Rα KO**	**WT vs. IL-18Rα KO**
iNOS	123.8 ± 12.8 vs. 12.9 ± 15.1 **	2.8 ± 3.2 vs. 2.2 ± 1.2
**M2 Macrophage**		
IL-4Rα	6.4 ± 1.7 vs. 2.4 ± 1.2 **	4.2 ± 1.3 vs. 4.7 ± 0.6
M1/M2 ratio	19.0 ± 4.0 vs. 4.2 ± 1.6 **	1.3 ± 0.5 vs. 1.3 ± 0.2
**Dendritic Cell**		
CCR7	9.8 ± 0.8 vs. 2.1 ± 0.7 ***	1.9 ± 0.8 vs. 1.0 ± 0.2

The M1/M2 macrophages’ phenotype gene expressions were measured in IL-18Rα KO and WT mice at 18 and 120 h after LPS injection by real-time PCR. In each experiment, the expression levels were normalized to the expression of 18SrRNA and are expressed relative to the values of saline-treated control mice. The data are the mean fold-increase ± SEM (** *p* < 0.01, *** *p* < 0.001). iNOS, nitric oxide synthase.

**Table 4:** Primer sequences for analysis of mRNA expression.

	Forward Primer	Reverse Primer
18SrRNA	GTAACCCGTTGAACCCCATTC	GCCTCACTAAACCATCCAATCG
TNF	CGATCACCCCGAAGTTCAGTA	GGTGCCTATGTCTCAGCCTCTT
CCL2/MCP-1	AAAAACCTGGATCGGAACCAA	CGGGTCAACTTCACATTCAAAG
GATA3	AGGGACATCCTGCGCGAACTGT	CATCTTCCGGTTTCGGGTCTGG
ROR^γt^	AGATTGCCCTCTACACG	GGCTTGGACCACGATG
T-bet	CCTGGACCCAACTGTCAACT	AACTGTGTTCCCGAGGTGTC

**Table 5:** Gene database number for analysis of mRNA expression.

	Gene Database No
18SrRNA	NM_026744.3
IFN-γ	NM_008337.3
IL-4	NM_021283.2
IL-6	Mm00446190
IL-10	NM_010548.1
IL-12p40	NM_008352.2
IL-18	NM_008360.1
IL-18R1 (IL-18Rα)	Mm00515180_m1
IL-18R2 (IL-18Rβ)	Mm00516053_m1
Kim-1	NM_134248.1
TLR4	Mm00445273_m1
CCR7	Mm99999130_s1
iNOS	Mm00440502
IL-4Rα	Mm01275139

## Supplementary Materials

The following are available online at www.mdpi.com/1422-0067/18/12/2777/s1.

## Abbreviations

AKI	Acute Kidney Injury
APCs	Antigen-Presenting Cells
BUN	Blood Urea Nitrogen
c/gcs	Cell Numbers Per Glomerular Cross-Section
c/hpf	Cell Numbers Per High-Power Field
CCL2	Chemokine (C-C Motif) Ligand 2
CCR7	C-C Chemokine Receptor Type 7
DCs	Dendritic Cells
IFN	Interferon
IL-18Rα KO	Interluekin-18 Receptor α Knock-Out
IL-18Rβ	Interluekin-18 Receptor β
IL-4Rα	Interleukin-4 Receptor Alpha
iNOS	Inducible Nitric Oxidase Synthase
Kim-1	Kidney Injury Molecule-1
LPS	Lipopolysaccharide
MCP-1	Monocyte Chemoattractant Chemokine-1
MHC	Major Histocompatibility Complex
TLR4	Toll-Like Receptor 4
TNF	Tumor Necrosis Factor
WT	Wild-Type
