# Supplementary material for: Lipopolysaccharide-Induced Acute Kidney Injury Is Dependent on an IL-18 Receptor Signaling Pathway
Source: Int J Mol Sci. 2017 Dec 20;18(12):2777. doi: 10.3390/ijms18122777 (PMC5751375; doi:10.3390/ijms18122777)
Supplement: Supplementary file 1 [file ijms-18-02777-s001.pdf]

International Journal of
Molecular Sciences

MDPI

Article

Lipopolysaccharide-Induced Acute Kidney Injury Is Dependent on an IL-18 Receptor Signaling Pathway

Supplementary Table 1. Clinical parameters of experimental mice.

	18 h	120 h
	**WT vs. IL-18Rα KO**	**WT vs. IL-18Rα KO**
MAP (mmHg)	88.8 ± 12.1 vs. 92.9 ± 18.2	93.2 ± 42.1 vs. 82.3 ± 16.8
HR (beats per minute)	400.4 ± 23.7 vs. 440.8 ± 31.2	386.2 ± 18.3 vs. 412.7 ± 29.6

We measured the blood pressure values of mice by a tail cuff at 18 and 120 h (each group; $n = 4$). The data are the mean ± SEM. Abbreviation; MAP, mean arterial pressure; HR, heart rate.

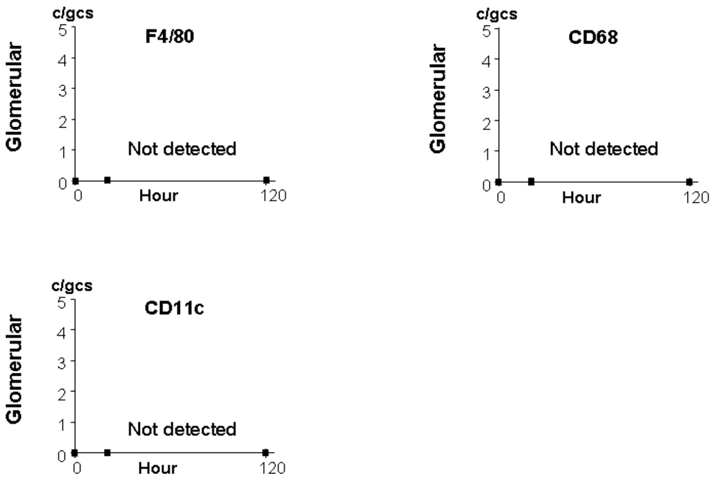

Supplementary Figure 1. Effect of IL-18Rα on the accumulation of inflammation cells in the glomerulus after LPS injection The accumulation of F4/80$^+$, CD68$^+$ and CD11c$^+$ cells in the glomerulus at 0 (KO and WT, $n = 3$), 18 ($n = 11$ and $n = 10$) and 120 h ($n = 16$ and $n = 6$) before and after LPS injection.

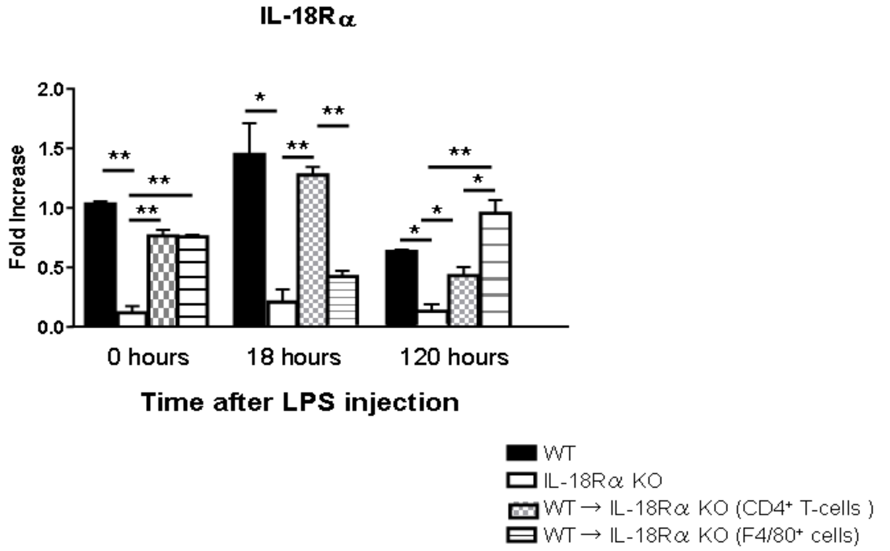

Supplementary Figure 2. Effect of splenocyte transfer on mRNA expression of IL-18Rα. Mice were sacrificed at 0, 18 and 120 h after LPS injection (each group; $n = 6$). Gene expressions of IL-18Rα were measured by real-time PCR. In each experiment, the expression levels were normalized to the expression of 18SrRNA and were expressed relative to the values of saline-treated control mice. The data are the mean fold-increase ± SEM: * $p < 0.05$, ** $p < 0.01$.
